# Bifocal Presentation of Primary Testicular Extranasal NK/T-Cell Lymphoma: A Case Report and Review of the Literature

**DOI:** 10.1155/2013/267389

**Published:** 2013-08-12

**Authors:** Ali Naboush, Firas Farhat, Selim M. Nasser, Francois G. Kamar

**Affiliations:** ^1^Staten Island University Hospital, NY, USA; ^2^Clemenceau Medical Center, Beirut, Lebanon; ^3^Division of Hematology & Oncology, Clemenceau Medical Center, Beirut, Lebanon

## Abstract

*Introduction*. Testicular lymphoma is an aggressive disease with a very poor prognosis. Nasal-type natural killer/T-cell lymphoma (NKTCL-N) in particular is very uncommon and has a rapidly progressive, fatal course. *Case Report*. We report a case of primary NKTCL-N of the testis in a 38-years-old Middle Eastern man. The patient had a history of primary right testicular tumor diagnosed at an outside institution as a seminoma and treated with orchiectomy followed by chemo/radiation. On admission, the patient had an enormous nasal granuloma with blood workup showing pancytopenia and elevated liver function tests due to active hepatitis B infection. CT scan of the sinuses showed a very large soft tissue mass, and PET scan showed splenomegaly with multiple lymph node masses in the pelvis and the chest areas. Bone marrow and nasal tumor biopsies as well as review of the slides from the initial orchiectomy were all in favor of NKTCL-N lymphoma. The patient was treated with CHOD based combination chemotherapy and responded dramatically to the first two cycles but passed away from fulminant hepatitis B infection. *Conclusion*. Despite all known treatments of NKTCL-N lymphoma of the testes, this disease has a very poor prognosis and invariably follows an aggressive clinical course.

## 1. Introduction

According to the World Health Organization (WHO) classification of hematolymphoid tumors, there are three categories of natural killer cell tumors, extranodal NK/T-cell lymphoma, the nasal type (NKTCL-N), an aggressive NK cell leukemia, and blastic NK cell lymphoma. The NKTCL-N lymphoma has two distinct variants, depending on the site of origin (nasal versus nonnasal) [1]. The Nasal variant occurs mainly in midline structures including the nasal cavity, nasopharynx, and paranasal sinuses, whereas the extranasal one involves the skin, the gastrointestinal tract, the salivary glands, the spleen, the lungs, and the testis [1]. Regardless of these presentations, the WHO classification groups both nasal and extranasal NKTCL-N lymphoma in the same category as “nasal type” although they have different clinical manifestations, treatment approaches, and prognosis [2, 3]. 

Primary testicular lymphoma (PTL) is a rare disease accounting for 1% of non-Hodgkin's lymphomas and is a potentially fatal disease. Diffuse large B-cell lymphoma (DLBCL) constitutes more than 70% of testicular lymphomas, occurring mostly in the seventh decade and has an aggressive clinical course [4]. Testicular NKTCL-N lymphoma is a very rare entity with an aggressive natural history with tumors cells expressing CD56. To date there are only 15 reported cases in the literature, and these mainly occurred in Asians and the Native American populations of Mexico and Central and South America [5–18]. In this case report we will report the 16th case of primary testicular NKTCL-N lymphoma and the first case to be reported in the Middle East. 

## 2. Case Report

A 38-years-old Middle Eastern man was admitted to our hospital for the management of a nasal granuloma. He had a history of primary right testicular tumor diagnosed at an outside institution as a seminoma and treated with orchiectomy followed by single agent Cisplatin and radiation therapy to the retroperitoneum. Few months after this diagnosis, the patient had an SMR procedure for nasal septal deviation. Shortly after that he developed nasal infection that progressed gradually into a very large granuloma. Despite a broad spectrum antibiotic coverage and antifungal therapy given at that institute, the patient nasal granuloma did not improve and was then associated with dysphagia and multiple oral ulcers. 

**Figure 1:**
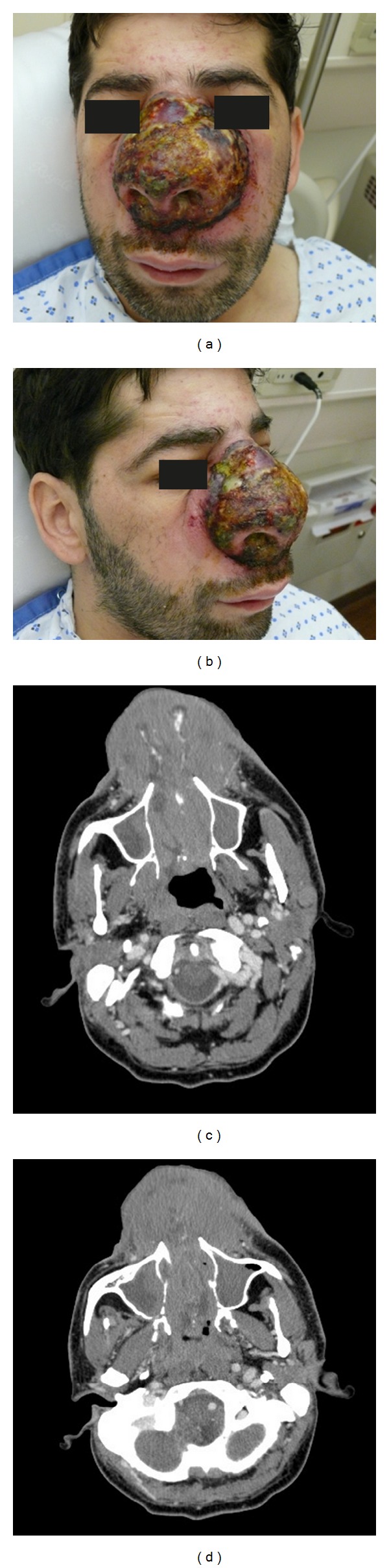
Pictures (a) and (b) showing anterior and lateral views of nasal granuloma from metastatic testicular ENKTCL-N lymphoma. Pictures (c) and (d) showing a CT of the sinuses with and without contrast, respectively, showing the nasal mass. Note: the patient had consented for the use of those pictures in research purposes.

Upon admission to the hospital, physical exam showed a very large nasal granuloma with swelling of the soft tissues of the face. In addition, he had necrotized crusty and discolored cutaneous lesions with abundant purulent discharge (Figure 1). Oral inspection revealed tonsillar, palatine, and uvular involvement with infected foul smelling discharge. Also the patient was noted to have obvious bulky inguinal lymph nodes that were fixed and hard, hepatosplenomegaly, and left lower extremity edema. 

Blood workup showed anemia, thrombocytopenia, and moderate lymphopenia as confirmed by peripheral smear. He also had elevated AST/ALT about ten times the normal upper limit, EBV IgG positive, and HbsAg positive in favor of active hepatitis B infection. Culture of the nasal swab grew pseudomonas and Candida. CT scan of the sinuses showed large 10 cm soft tissue mass in the nose extending into the nasal cavity and necrotic lesions in the tonsils (Figure 1). PET scan showed splenomegaly, multiple lymph node masses in the pelvis, tracheal, and laryngeal walls. 

**Figure 2:**
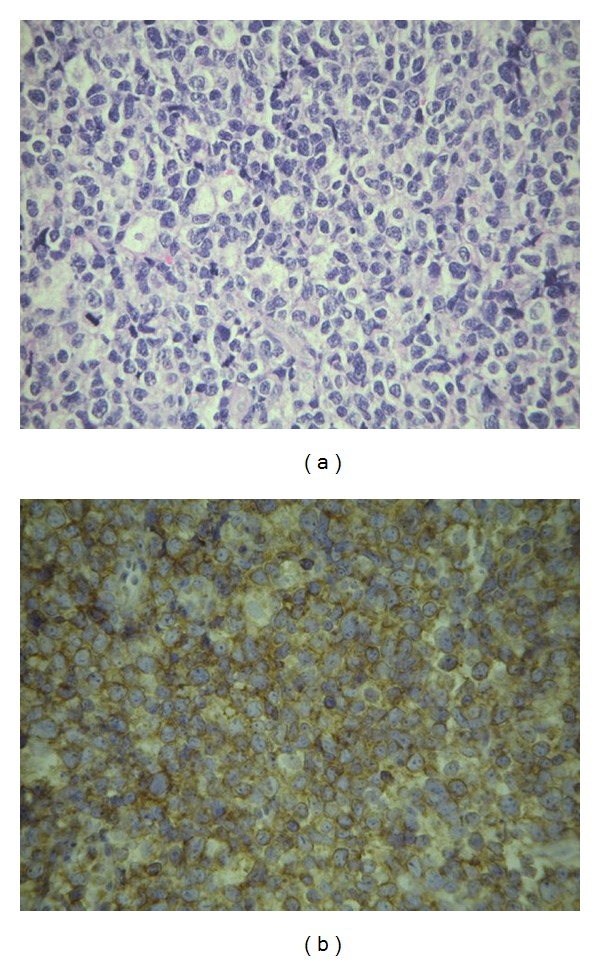
Picture (a) showing dense and diffuse polymorphous population of tumor cells consisting of small to medium sized and large cells with irregular nuclei. Picture (b) showing an immunohistochemistry with tumor cells expressing CD56.

Bone marrow and nasal tumor biopsies revealed dense and diffuse proliferation of medium to large sized atypical lymphoid population (Figure 2(a)). The tumor cells expressed LCA, CD56, and CD3 cytoplasmic and were negative for CD20 suggesting NKTCL-N lymphoma (Figure 2(b)). Review of the slides from the initial orchiectomy was also diagnostic of primary testicular NKTCL-N. 

The patient was treated with Imipenem and Caspofungin for his local superinfection until he improved. He was then started on Lamivudine and Interferon alpha-2b as a treatment for hepatitis B prior to discharge from the hospital. Preventive measures for tumor lysis syndrome were then started, and the patient was offered a CHOD based combination chemotherapy. The patient responded dramatically and rapidly to the first two cycles with repeat CT scan of the total body showing near total resolution of the lymphadenopathies. He had also a near total regression of the nasal lesion. Unfortunately, the patient passed away from fulminant hepatitis B infection. 

## 3. Discussion

Primary testicular ENKTCL-N lymphoma is a very rare disease with only 15 cases reported in the English literature [5–18]. Those cases were collected through a MEDLINE search performed from 1965 to 2013 using keywords like NK T-cell lymphoma, testicular, and nasal type. The average age of occurrence was 49.4 (ranging from 28 to 76). Eight of the reported cases were described in Asian people, one in a Hispanic Columbian and one in a Caucasian French with the others not identified, consistent with the prevalence of NKTCL-N lymphoma in those ethnic groups (except for the Caucasian one) [11]. Among the 15 cases, the disease relapsed mainly to the contralateral testis and lymph nodes (5 and 4 cases, resp.) and to a lesser degree to the spleen, skin, liver, and CNS (3, 3, 2, 2 cases, resp.). Only one of the patients had his disease relapsed to the nasopharynx [15]. Almost all patients underwent an orchiectomy and were treated after that with a CHOP based chemotherapy regimen and intrathecal Methotrexate followed by radiation therapy. Despite this multimodality approach, the majority of patients were dead within 6 months except for 4 who were alive at 9–14 months but with no further follow-up period reported [11]. 

This is the first case of primary testicular NKTCL-N in a Middle Eastern patient to be reported and the second case identified in a Caucasian patient [11]. It is also the second reported case to metastasize to the nasal cavity [15]. The characteristics of the NK/T-cell lymphoma in our patient were typical including EBV positivity and tissue biopsy showing dense and diffuse proliferation of medium to large sized atypical lymphoid population expressing LCA, CD56, and CD3 cytoplasmic and were negative for CD20 [5–18]. 

Testicular ENKTCL-N is very uncommon and has a rapidly progressive, fatal course. The association of EBV infection contributes to the increased risk, which adversely affects patient survival [14], suggesting that the presence of EBV may correlate closely with prognosis in primary NKTCL-N lymphoma of the testis. Despite intensive chemotherapy treatments, this disease is highly aggressive with an early skin, aerodigestive tract, or soft tissue involvement. Almost all patients whose lesions seem to be limited to the testis relapse within 6 months and die of widespread dissemination to the skin, CNS, GI tract, or lung within 1 year. 

## 4. Conclusion

Primary testicular NKTCL-N is an aggressive type of lymphoma with very poor prognosis that invariably follows an aggressive clinical course irrespective of gender and the kind of treatments used.
